# Fish with Chips: Tracking Reef Fish Movements to Evaluate Size and Connectivity of Caribbean Marine Protected Areas

**DOI:** 10.1371/journal.pone.0096028

**Published:** 2014-05-05

**Authors:** Simon J. Pittman, Mark E. Monaco, Alan M. Friedlander, Bryan Legare, Richard S. Nemeth, Matthew S. Kendall, Matthew Poti, Randall D. Clark, Lisa M. Wedding, Chris Caldow

**Affiliations:** 1 U.S. National Oceanic and Atmospheric Administration, Center for Coastal Monitoring and Assessment, Silver Spring, Maryland, United States of America; 2 Center for Marine and Environmental Studies, University of the Virgin Islands, St. Thomas, Virgin Islands, U.S. Virgin Islands; 3 Centre for Marine and Coastal Policy Research, The Marine Institute, Marine Building, Plymouth University, Plymouth, Devon, United Kingdom; 4 Department of Biology, Fisheries Ecology Research Laboratory, University of Hawai‘i at Mānoa, Honolulu, Hawai'i, United States of America; 5 Texas Parks and Wildlife, Dickinson Marine Lab, Dickinson, Texas, United States of America; 6 Center for Ocean Solutions, Woods Institute for the Environment, Stanford University, Monterey, California, United States of America; The Australian National University, Australia

## Abstract

Coral reefs and associated fish populations have experienced rapid decline in the Caribbean region and marine protected areas (MPAs) have been widely implemented to address this decline. The performance of no-take MPAs (i.e., marine reserves) for protecting and rebuilding fish populations is influenced by the movement of animals within and across their boundaries. Very little is known about Caribbean reef fish movements creating a critical knowledge gap that can impede effective MPA design, performance and evaluation. Using miniature implanted acoustic transmitters and a fixed acoustic receiver array, we address three key questions: How far can reef fish move? Does connectivity exist between adjacent MPAs? Does existing MPA size match the spatial scale of reef fish movements? We show that many reef fishes are capable of traveling far greater distances and in shorter duration than was previously known. Across the Puerto Rican Shelf, more than half of our 163 tagged fish (18 species of 10 families) moved distances greater than 1 km with three fish moving more than 10 km in a single day and a quarter spending time outside of MPAs. We provide direct evidence of ecological connectivity across a network of MPAs, including estimated movements of more than 40 km connecting a nearshore MPA with a shelf-edge spawning aggregation. Most tagged fish showed high fidelity to MPAs, but also spent time outside MPAs, potentially contributing to spillover. Three-quarters of our fish were capable of traveling distances that would take them beyond the protection offered by at least 40–64% of the existing eastern Caribbean MPAs. We recommend that key species movement patterns be used to inform and evaluate MPA functionality and design, particularly size and shape. A re-scaling of our perception of Caribbean reef fish mobility and habitat use is imperative, with important implications for ecology and management effectiveness.

## Introduction

In the past 50 years, coral reef ecosystems of the Caribbean Sea have experienced considerable region-wide declines in the abundance and body size of many fished species and in the quality of their habitat [1], [2], [3]. In response, marine protected areas (MPAs), including areas closed to fishing, have been established throughout the region [4]. However, MPAs often fail to reach their full potential as a consequence of factors such as illegal harvesting, regulations that legally allow detrimental harvesting, or emigration of animals outside boundaries because of continuous habitat or inadequate size of the MPA [42]. In the Caribbean and elsewhere, MPA placement, size and shape, typically have been delineated without knowledge of the movements of the animals they are intended to protect. This is a widespread practice for coral reef-centered MPAs where placement and shape are generally designed using relatively static biophysical features of the seafloor and with political and socio-economic considerations rather than criteria based on functional ecology [32], [33], [43], [44]. Research on the optimal design and connectivity of MPAs and networks of MPAs has focused primarily on models of larval dispersal [5], [13], [37]. It is now increasingly acknowledged, however, that adult and juvenile movements have a considerable influence on the functioning of MPAs [7], [34], [40], [41]. Spatially-explicit information on the movement patterns of fishes, including home range size, relocation movements and distances traveled on migrations, can play a central role to support the ecologically meaningful design of MPAs [5], [6], [10], [45]. For highly mobile fishes, protection could be sub-optimal if MPAs are too small relative to the routine space use patterns of fishes, or if placed in locations that offer incomplete protection for animals when undertaking critical migrations [5], [6], [7], [13]. Modeling studies suggest that even limited adult dispersal can have a large impact on MPA functioning [44]. Conversely, emigration from protected areas can contribute to “spillover” of fish into the neighboring fishery, an important fisheries management goal [8]. Clearly, knowledge of the spatial scale and patterns of fish movements across the seascape can provide direct evidence of ecological connectivity between individual MPAs, as well as data for the evaluation of ecological coherence within an MPA network.

Understanding movement patterns has profound implications for ecology too. Organism movement behavior is a fundamental ecological process that can be used to identify habitat use patterns, investigate bioenergetics, energy flows and to select the appropriate spatial (and temporal) scale(s) at which to study species ecology [9], [11]. An organism-centered approach, that provides explicit spatial and temporal data on organism movements, can help ecologists formulate research questions and design studies that measure ecological patterns and processes at ecologically meaningful scales [9]. When analyzed together with maps of benthic composition, or seafloor topographic structure, movement data can identify critical pathways, or ‘blue corridors', connecting patch types in the seascape [9], [10], [11], [12]. Very little is known, however, about the spatial scale of adult fish movements, travel times and locations visited by Caribbean fishes during their life span [7], [9].

Fish assemblages of Caribbean coral reef ecosystems are exceptionally diverse, with many species using multiple habitat types (i.e., seagrasses, mangroves, coral reefs) through daily home range movements, ontogenetic habitat shifts and spawning and non-spawning migrations [9], [14]. As such, the spatial domain of routine movements, sometimes referred to as the ecological neighborhood [9], [10], will differ between species, individuals and life-stages [6], [14], [15]. However, direct and spatially explicit evidence for the distances traveled and timing of movements by fishes associated with Caribbean coral reef ecosystems is scarce [16]. Studies have either focused on fine-scale movements for single species and families, usually confined geographically to a single embayment or MPA [19], [20], or large-bodied species of high conservation or fishery concern, such as groupers, revealing long (>30–220 km) distance movements to spawning aggregations [35], [36], [49]. Relative to the fish life span, movement observations have been short (days-weeks) potentially resulting in an underestimate of the spatial scale of movement [7].

The most effective technique for gathering direct evidence of movements in time and space for highly mobile marine animals, such as fish, is to tag them with miniature acoustic transmitters [9], [21]. Advances in microelectronics and a demand for ever smaller tags has resulted in the development of miniaturized underwater acoustic transmitters small enough to be harmlessly implanted in small (<20 cm) fishes, yet with sufficient battery life to emit a unique identification code every minute for a year or more. These coded transmitters are detected by underwater acoustic receivers that can be strategically moored above the seafloor to form an array of listening stations [22].

A lack of information on fish movements has been identified as a critical knowledge gap in the application of MPAs as a management tool [17], [18], [34]. To address the knowledge gap for the U.S Caribbean we utilized an array of 225 underwater acoustic receivers to track fish movements between MPAs and across the contiguous insular shelf of the U.S. Virgin Islands and southeastern Puerto Rico. The array known as the U.S. Caribbean Acoustic Network (USCAN), formed through a consortium of collaborating scientists from multiple institutions, was one of the largest acoustic fish telemetry networks in the world. Tracking movements of highly mobile organisms with an acoustic array allowed us, from afar, to locate fish and then map points of presence along their movement pathways as they swam freely across the seascape.

Our primary research questions were: 1.) How far do fish move and how long does it take them to move those distances? 2.) Are MPAs connected by fish movements and if so then how often do fish cross MPA boundaries? and 3.) Are MPAs in the eastern Caribbean of sufficient size to encompass the movements of reef fish?

## Materials and Methods

We tagged 184 individual fish with surgically implanted miniature acoustic transmitters (see File S1) representing 19 species from 10 different families commonly occurring in Caribbean coral reef ecosystems (Table 1) [23]. Tagged fishes included ecologically and economically important species targeted in the Virgin Islands fishery. Fish sizes, measured as total body length (TL), ranged from 19 to 70 cm (mean = 30.3 cm). The largest individual tagged was a 70 cm TL nurse shark (*Ginglymostoma cirratum*) and the smallest was a blue tang (*Acanthurus coeruleus*) of 19 cm TL. Five species of snapper (Lutjanidae) accounted for 31% of all individuals tagged and three species of grunt (Haemulidae) accounted for 23% of the sample. At the species level, bluestriped grunt (*Haemulon sciurus*) comprised 22% of all individuals tagged, lane snapper (*Lutjanus synagris*) 21%, bar jack (*Caranx ruber*) 11%, and saucereye porgy (*Calamus calamus*) 10%.

**Table 1:** Fish species tagged with ultrasonic transmitters and their biological characteristics and summary information on the duration of tracking and least cost distance between farthest receivers with detections.

Family	Scientific name	Common name	Number tagged	Trophic group	Total length range (cm)	Max. days tracked	Max inter-receiver distance (range in km)
Haemulidae	*Haemulon sciurus*	Bluestriped grunt	40	Invertivore	24–30.6	930	0.4–13.5
Lutjanidae	*Lutjanus synagris*	Lane snapper	38	Inv/Pisc	20–36	722	0.5–11.5
Carangidae	*Caranx ruber*	Bar jack	21	Piscivore	29.8–47	329	0.58–13.6
Sparidae	*Calamus calamus*	Saucereye porgy	19	Invertivore	21.3–35	180	0.47–15.3
Lutjanidae	*Ocyurus chrysurus*	Yellowtail snapper	14	Invertivore	22.5–38	333	0.3–16.1
Lutjanidae	*Lutjanus analis*	Mutton snapper	12	Invertivore	31–65	784	1.1–42.2
Holocentridae	*Holocentrus adscensionis*	Longjaw squirrelfish	9	Invertivore	26–29	348	1.5–5.1
Acanthuridae	*Acanthurus coeruleus*	Blue tang	5	Herbivore	19–24	106	2–8.5
Balistidae	*Balistes vetula*	Queen triggerfish	5	Invertivore	29–39	38	0.58–1.5
Lutjanidae	*Lutjanus griseus*	Gray snapper	5	Invertivore	25.2–35.4	657	1–3.5
Mullidae	*Mulloidichthys martinicus*	Yellow goatfish	3	Invertivore	31–32.3	34	0.4–4.8
Acanthuridae	*Acanthurus chirurgus*	Doctorfish	2	Herbivore	19.2–23.9	85	1–3.6
Serranidae	*Epinephelus guttatus*	Red hind	2	Inv/Pisc	29.5–36.5	402	6.3
Ginglymostomatidae	*Ginglymostoma cirratum*	Nurse shark	2	Inv/Pisc	55–70	233	0.7
Haemulidae	*Haemulon flavolineatum*	French grunt	2	Invertivore	20–21.5	1	0
Haemulidae	*Haemulon plumierii*	White grunt	2	Invertivore	25–31.5	332	6.2
Lutjanidae	*Lutjanus apodus*	Schoolmaster	1	Inv/Pisc	27	382	5.1
Lutjanidae	*Lutjanus jocu*	Dog snapper	1	Inv/Pisc	41.4	311	12.8
Mullidae	*Pseudupeneus maculatus*	Spotted goatfish	1	Invertivore	27	0	0

Note that these estimates represent the minimum known cross-boundary movements. We except that the maximum distance estimates are likely to be limited by the array configuration and that many movements are undetected, but this only increases the probability that distances traveled could be even greater than highlighted here rather than less extensive.

Fish were captured, tagged and released between July 2006 and July 2008 within the Virgin Islands National Park (VINP) under research permit (# 118794) provided by the National Park Service of the U.S. Department of Interior. Fish locations were recorded for a total of 1,244 days (approx. 3 year and 4 months) calculated as the time elapsed between the first and last detection on a receiver. During that time, a total of 2,848,192 detections were recorded from 163 individual fish of 18 species (21 individuals were not detected after release).

Experimental procedures were approved by the Office of Research Compliance Animal Welfare and Biosafety Program of the University of Hawaii and the Animal Care and Use Committee. Permission to capture fish and conduct experiments including tagging and tracking inside waters managed by the U.S. Department of Interior were provided by the National Park Service under permit NPS PIMS Project #118794 Ecological linkages between the Virgin Islands Coral Reef National Monument and the Virgin Islands National Park.

### Acoustic array

**Figure 1:**
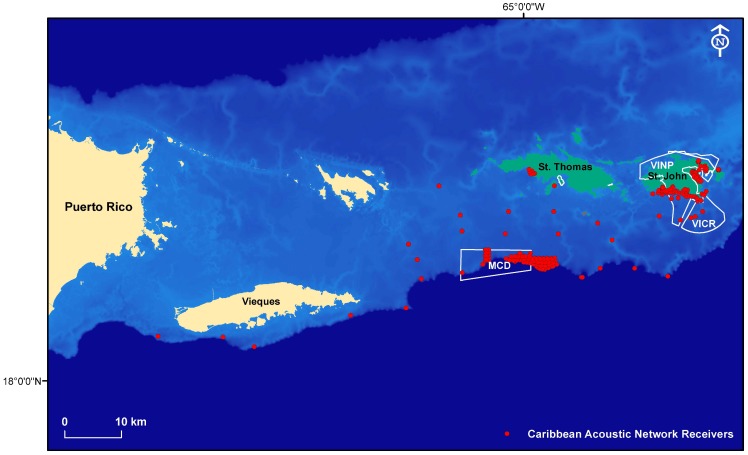
Location of underwater acoustic receivers in the U.S. Caribbean Acoustic Network (USCAN) on the southern insular shelf of Puerto Rico and U.S. Virgin Islands. MPA boundaries are shown for the Virgin Islands National Park (VINP), Virgin Islands Coral Reef Monument (VICR) and the Marine Conservation District (MCD).

The underwater acoustic monitoring system consisted of an array of Vemco™ VR2/VR2W acoustic receivers (340 mm long 960 mm diameter, weight in water 300 g) capable of detecting coded transmitter signals when located within a 300 m minimum radius of the receiver. Receivers were deployed on polypropylene lines or vinyl coated stainless steel wire attached to the seabed with sand screws or concrete blocks at depths of between 1 and 38 meters. The array was positioned close to the edges of nearshore coral reefs and across the insular shelf to create both an alongshore and an inshore-offshore corridor of listening stations (Fig. 1). Receivers were located both inside and outside four well-established, permanent and seasonal marine protected areas of the U.S. Virgin Islands: Virgin Islands Coral Reef National Monument (VICR) (approx. 51.5 km^2^); the Virgin Islands National Park (VINP) (approx. 22.9 km^2^); Hind Bank Marine Conservation District (MCD) (approx. 41 km^2^), and Grammanik Bank (GB) (approx. 4.5 km^2^), the latter two being sites of regionally important multispecies fish spawning aggregations. VICR and MCD are both no-take MPAs and GB is a seasonal closure for a range of spawning species [50]. Our array also provided information on trans-boundary movements from waters managed by U.S. Federal Government to waters managed by the Governments of Puerto Rico and U.S. Virgin Islands. Receivers were maintained and data managed and shared by a consortium of institutions known collectively as the U.S. Caribbean Acoustic Network (USCAN) comprising the National Oceanic and Atmospheric Administration's (NOAA) National Centers for Coastal Ocean Science (NCCOS) and NOAA National Marine Fisheries Service (NMFS), together with the University of the Virgin Island's Center for Marine and Environmental Studies. When operating simultaneously, this network was able to detect tagged fish movements between receivers across distances of between 300 m (closest receivers) to 103.3 km (farthest receivers) across the insular shelf. Although the integrated data from the entire array network provided greater coverage than could any of the individual project arrays, some spatial clustering of receivers resulted from arrays configured to address specific research questions.

### Calculating least-cost pathways between acoustic receivers

To quantify the spatial domain of movements for each fish, we calculated between- receiver distance for all receivers that detected a fish and reported the distance between the pair of receivers that were farthest apart, together with shortest time elapsed between those detections. The distances we present here provide estimates of maximum known movement capability to define a spatial domain of space use for fishes. We select these data to address specific questions about the spatio-temporal scale of movements and we do not herein address detailed habitat use patterns. Rather than calculating straight line distances between each pair of receivers, some of which would have transected land or deep waters beyond the shelf edge, we quantified distances from modeled least-cost pathways (LCP) conditioned to follow suitable habitat for reef fish (i.e. colonized hardbottom habitat and shallow water non-reef habitat [sand]) while avoiding less suitable or unsuitable environments (i.e., land and deep water beyond the shelf edge) (see File S1). Tracking studies and underwater observations provide evidence that fish follow coral reefs to maximize access to food and minimize predation risk, whereas land and deep water and sometimes large expanses of sand can function as barriers to movement [7], [24], [39]. Non-reef sandy areas were weighted with relatively low cost such that pathways could cross sand to connect patches of more preferred (lowest cost) coral reef patches. Parameterization of LCP models was based on the known daytime occurrence of species over shallow water coral reef ecosystems where a strong dependence exists for colonized carbonate seafloor which provides appropriate refuge from predation and potential food resources for the traveling fish.

### Quantifying movements across MPA boundaries

To examine movements relative to MPA boundaries, we calculated the number of times each fish crossed a management boundary including excursions to unprotected areas outside of MPAs. Fish that did not cross a boundary and appeared to remain within the VINP during the tracking period were also quantified. Trans-boundary movements were recorded between the Virgin Islands National Park (limited fishing restrictions); Virgin Islands Coral Reef National Monument (no-take); Hind Bank Marine Conservation District (no-take spawning aggregation); Grammanik Bank (seasonal closure spawning aggregation/pelagic fishing only) and non-MPA waters. Detections from receivers that were located too close to a boundary to provide high certainty that a fish had crossed a boundary were removed from this part of the analysis. This resulted in removal of seven receivers along the VINP-VICR boundary and a large spatial gap in detection, therefore, our estimates of boundary crossings should be interpreted as a minimum of crossings.

### Estimating the proportion of tracked time fishes spend inside and outside MPAs

To examine the presence and residence times of individual fish inside and outside MPAs, a Fidelity Index was calculated as a relative estimate of minimum residency. Individuals were considered present if two or more detections occurred on at least one receiver within a given day. Fishes were excluded from analysis where less than a total of ten detections were recorded across the monitoring period or where presence was recorded for only one day or less. The monitoring period for each fish was defined as the number of days between the tagging event and the last detection. Hourly fish presence was calculated by pooling detections into hourly increments (24 per day), such that if a fish was detected in an MPA or outside between 00:00 and 00:59 of any hour then it was considered present. If a fish was present in two zones in the same hour it received a value of 1 hour for each zone.

The proportion of time a fish was observed during the study was calculated as the hours present in all zones divided by the total monitoring period. To understand the preference for each zone (i.e. VICR, VINP and unprotected area), a location-specific Fidelity Index (FI) was quantified for each fish as the sum of hours in each zone divided by the total hours present. The proportion of time represented in the FI ranged from 0 (no fidelity) to 1 (high fidelity).

Due to uncertainty in fish locations during periods without detections, the FI is best interpreted as the minimum amount of time a fish is present in a zone and can be used to understand the relative preference between zones, rather than absolute time spent in each zone.

### Quantifying spatial dimensions of eastern Caribbean MPAs

To calculate the spatial dimensions of MPAs (n = 220) throughout the Leeward and Windward Islands (U.S. Virgin Islands to Aruba, Bonaire and Curacao) we applied a GIS tool called Minimum Bounding Geometry using ArcGIS v10.1 (ESRI Inc, 2012) to quantify the length of the long axis (referred to as MPA length) and the perpendicular axis (referred to as MPA width) for each MPA polygon. To estimate the greatest distance that a fish inside an MPA would need to travel to reach the MPA boundary, we calculated the farthest-edge to centroid distance (the *apothem* for regular polygons) for each MPA polygon by halving the length and width dimensions. Spatial data on MPAs was provided by The Nature Conservancy compiled for the Caribbean Marine Protected Areas Managers Network and Forum (CaMPAM). The MPA database was in development at the time of writing and therefore incomplete and the regulations associated with each MPA were not available to determine the proportion of MPAs designated as no-take marine reserves. Because of differences in the way that fish pathways and MPA axes were measured we do not statistically compare fish distances traveled versus MPA length and width, but instead visually approximate the potential scale mismatch between spatial domains of MPAs and fish movement capability. In addition, differences in seascape configuration between individual MPAs preclude any further comparison.

## Results and Discussion

### How far can Caribbean reef fishes move?

**Figure 2:**
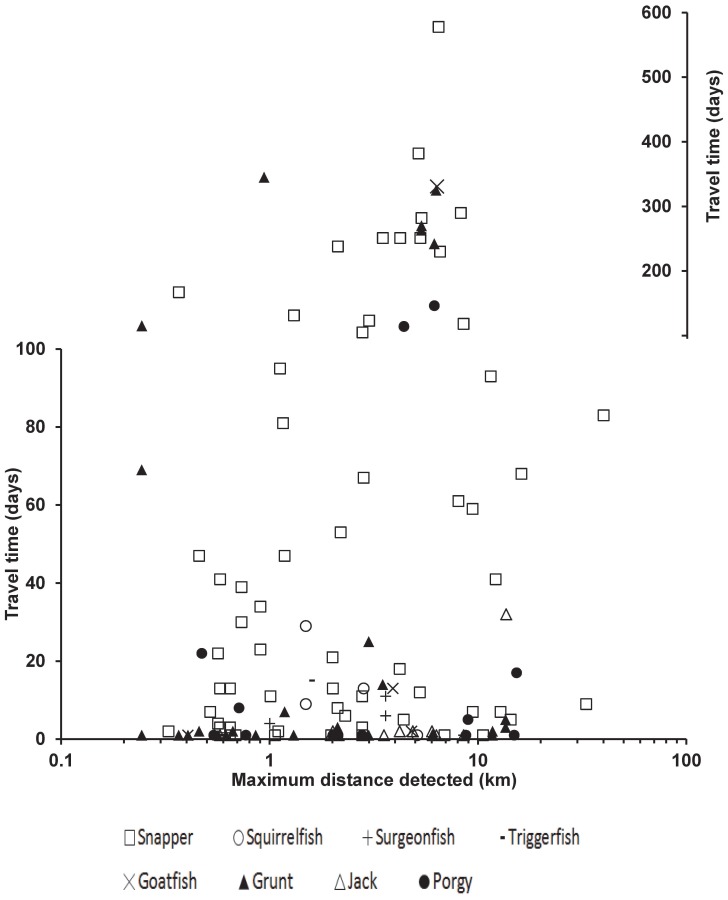
Distance and travel time between farthest receivers visited by acoustically tagged fish based on the time stamps of detections on the Caribbean acoustic array. Symbols identify fish family for0 each individual fish data point.

Our long-term acoustic tracking data extend considerably the known movement abilities for Caribbean reef fish (Fig. 2). The mean tracking duration calculated from all tagged individuals was 164 days (±15.6 SE), with a maximum detection period of 967 days for a bluestriped grunt (*Haemulon sciurus*), exceeding the manufacturer's estimate of battery life for the transmitters. Other long duration tracking periods were 722 days for a lane snapper (*L. synagris*); 784 days for a mutton snapper (*L. analis*); 657 days for a gray snapper (*L. griseus*) and 402 days for a red hind (*Epinephelus guttatus*) (Table 1).

From a total of 163 fish tracked, the estimated mean between-receiver distance traveled was 3.8 km (median = 2.1 km). Seventy-five percent of fish undertook long range movements greater than 1 km and 33% more than 5 km. Twenty-eight individual fish traveled more than 1 km between two receivers in a single 24 hour period. Three fishes traveled 14.9 km (*Calamus calamus*, 24.5 cm TL); 11.7 km (*Haemulon sciurus*, 30 cm TL); and 10.6 km (*Lutjanus synagris*, 30.4 cm TL) in a single day. At the family level, grunts (haemulids), snappers (lutjanids), jacks (carangids), and porgies (sparids) showed the greatest long-range movements. Unexpectedly high mobility, however, was also detected for several species that are usually perceived as exhibiting relatively low mobility highlighting how little is known about reef fish movements. For instance, during a detection period of 348 days, a longjaw squirrelfish (*Holocentrus adscensionis*) was detected on receivers separated by an estimated least-cost path distance of 5.1 km. In addition, we include information on species with little previous information on distance travelled, including an individual blue tang (*Acanthurus coeruleus*) with maximum estimated distance of 8.5 km; a yellow goatfish (*Mulloidichthys martinicus*) 4.8 km and 1.5 km for a Queen triggerfish (*Balistes vetula*). In contrast, eleven fish were only detected within 500 m of their first detection point during an average period of 40 days of tracking (range = 1–167). Longer range movements, however, may have occurred out of range of our receiver array. For example, in a conventional tag-recapture study red hind migrated from 5 to 33 km from spawning aggregation sites to home range sites [51], [52]. The modeled estimates of movement distances should be interpreted as best-available information on the spatial dimensions of the activity spaces for individual fish and an underestimate of the true distances moved within the life span due to spatial bias in the detectability of fish inherent with a fixed acoustic array. Furthermore, quantification of model uncertainty for least cost paths modeled on minimum cost for movements across a benthic cost surface requires further validation with detailed animal movement trajectories [48].

### Association between fish body size and spatial scale of movements

Several reviews of home range movements have found large-bodied reef fishes often have larger home ranges than smaller bodied fishes [6], [17]. After outliers were removed (fish detected only on a single receiver and the shark), our data demonstrate that body size (TL) and maximum distance of detection are positively and significantly correlated (*r* = 0.48, *p* = <0.05). Several large-bodied snapper which undertook long-distance migrations to shelf-edge spawning sites strengthened the positive correlation, but the correlation was also decoupled by high variability among individual fish together with highly variable tracking duration (Table 1). These differences in mobility are likely to be related to morphological and life-history characteristics, as well as the distribution of habitat structure across the region they inhabit. High behavioral polymorphism in movement patterns among fish species, life stages and individuals of the same species presents a major challenge in MPA network design, because over time unequal protection will exert a selection pressure for certain life history strategies [5]. In general, larger-bodied reef fishes are highly mobile, such as many large snappers, groupers and jacks that may undertake ontogenetic habitat shifts and spawning migrations across the shelf [38]. These broad-scale movements are thought to have a large impact on MPA functioning [5], [10], [17]. For example, Edgar et al. [42] found that MPA size was important for predicting biomass of jacks (Carangidae), a highly mobile family, whereby smaller MPAs correlated with lower biomass which was thought to result from increased time spent outside of park boundaries.

As often is the case with remote sensing approaches like acoustic telemetry, we are unable to provide information on the drivers of the movements that we report here. With a diverse range of species and families comes a diverse range of movement strategies. Some movements will be ontogenetic habitat shifts, others will be nomadic movements to find more optimal habitat, or enlargements of the home range, or very directional migratory movements to spawning aggregations. To adequately protect a range of species within an MPA or network of MPAs, the movement patterns for adult fish from a range of fished species needs to be considered in the MPA network design process [7], [9], [10]. Although our study provides new information for several species, longer duration continuous tracking of multiple individuals at different life stages, together with high resolution mapping of the seafloor will help to better understand the movement ecology of fishes associated with Caribbean coral reefs.

### Connectivity within an MPA network

A total of 278 confirmed boundary crossings were recorded during the study period by 12 of the 18 species. Twenty-five percent of individuals crossed one or more MPA boundaries in the U.S. Virgin Islands network of MPA's. Five percent (7 individuals of 4 species) crossed two or more different management boundaries. Sixteen percent (24 individuals of 11 species) crossed the boundary between the VINP and the VICR. The highest number of crossings (222 crossings from 21 individuals and 11 species) occurred between VINP and areas outside of any MPA, with only two crossings from VICR to outside areas. Fifty-four crossings (24 individuals of 11 species) were recorded between VINP and VICR confirming the existing of ecological connectivity between adjacent MPA units.

**Figure 3:**
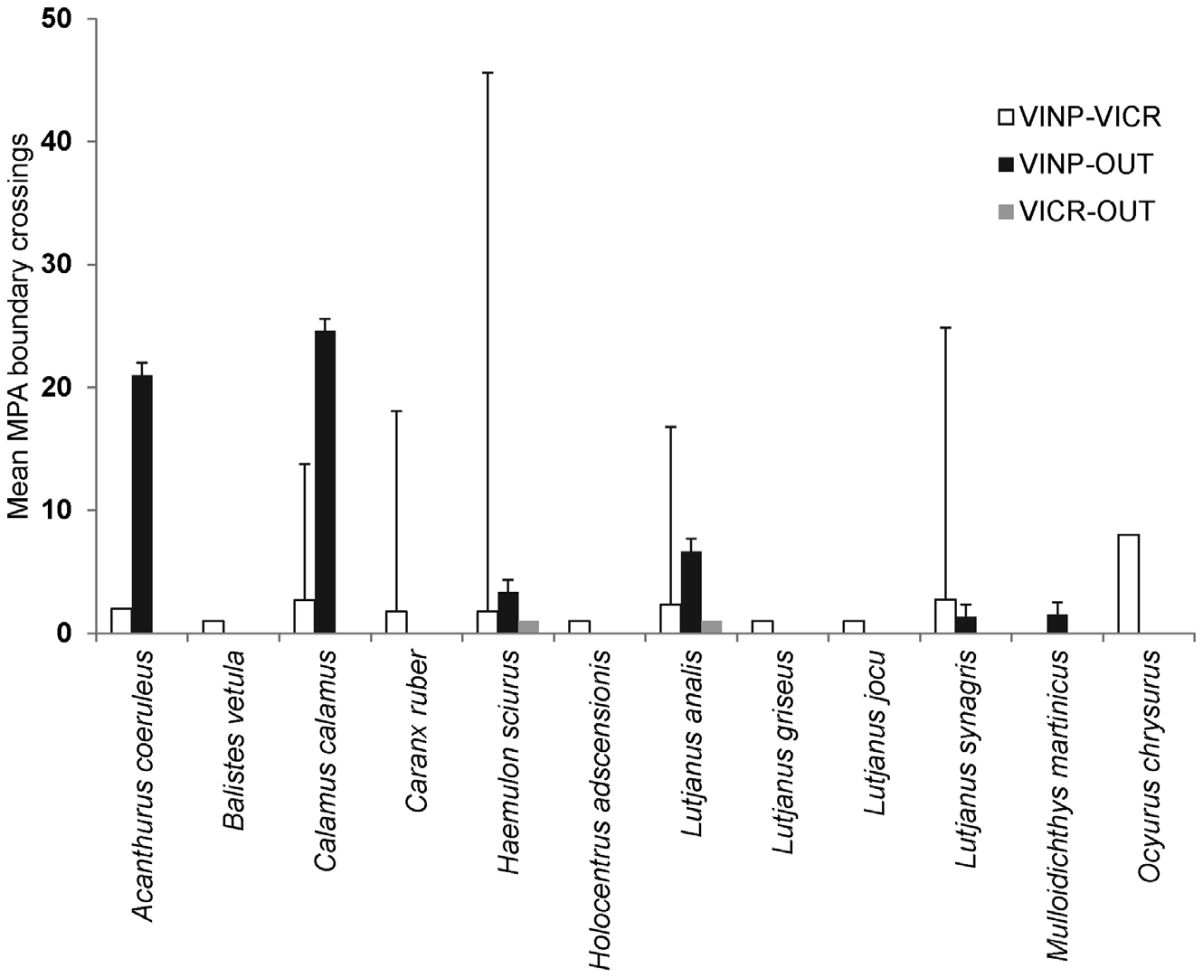
Mean (± SE) number of times individuals of a fish species crossed a management boundary (VINP – Virgin Islands National Park; VICR – Virgin Islands Coral Reef National Monument; Out – outside MPA) in the U.S.Virgin Islands (based on receiver detections) during the study period. Individuals from 12 of 18 species were confirmed to have crossed a management boundary.

Blue tang (*A. coeruleus*) bluestripped grunts (*H. sciurus*), four snapper species (*Lutjanus synagris, L. griseus, L. analis and Ocyurus chrysurus*), a jack species (*Caranx ruber*) and porgies (*Calamus calamus*) undertook most boundary crossings (Fig. 3). Many of these boundary crossings probably occurred because routine daily home range areas straddled the boundary of two adjacent MPAs or during a spawning migration. For instance, distinct day and night activity spaces sometimes occur in geographically discrete locations that can involve movements from deep to shallow habitat or from reef to seagrass beds [15], [20]. From a management perspective this means that many of the tagged fishes spent time in management units providing different levels of protection from fishing, with uncertain effects on MPA performance. The siting of a fully protected MPA adjoining a partially protected MPA could result in a net flow of organisms into the fully protected MPA, especially if that MPA provides higher habitat quality. Furthermore, connectivity, particularly for herbivorous fishes, has been found to boost MPA performance and increase reef resilience [40], [46], [47], presenting additional ecological consideration for the relevance of fish movements in the MPA design process.

**Figure 4:**
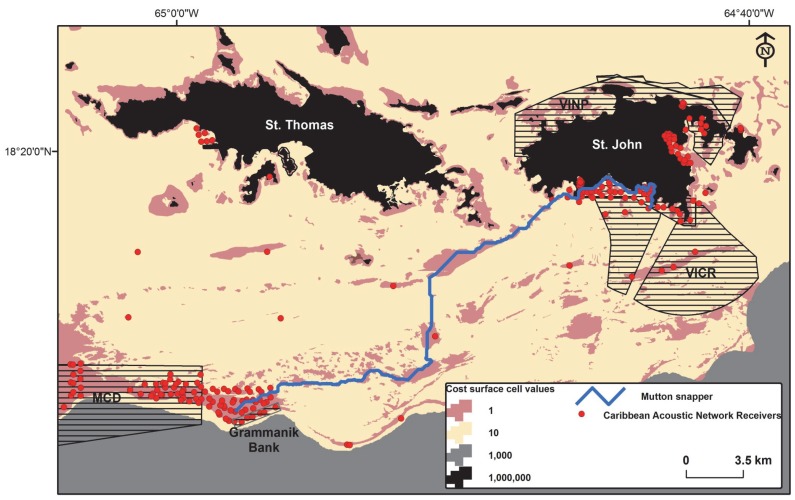
Cost surface for the U.S. Virgin Islands based on relative environmental suitability for shallow-water reef fish. One modeled least-cost path is shown for an individual mutton snapper (*Lutjanus analis*) that moved a maximum of 40.2 km between two receivers (Virgin Islands National Park [VINP] to shelf edge spawning aggregation). The path was parameterized to preferentially follow wherever possible the low cost coral reef to achieve a minimum accumulative travel cost between the two farthest receivers with detections.

One individual mutton snapper traveled from the nearshore seascapes of VINP to the shelf edge (Grammanik Bank MPA) and back through non-MPA waters providing new direct evidence of ecological connectivity between multiple neighboring MPAs and between near-shore protected areas and known shelf edge spawning aggregations (Fig. 4). The modeled path distance between farthest receivers for this long-distance migration was estimated at 40.2 km. Our study has demonstrated that fish tagged within MPAs in the U.S. Virgin Islands spend time (and use resources) under differing management regimes that offer different levels of protection from fishing. Furthermore, we provide confirmation that fishes tagged in protected areas also spend time in areas open to fishing. Therefore we demonstrate that fish do swim in and out of MPAs in the U.S. Virgin Islands. Even if only temporary excursions to areas outside MPAs, these fishes contribute to a ‘spillover’ process influencing biodiversity, productivity and ecological functions in neighboring unprotected areas [25], [26]. Although we conclude that ecological connectivity exists between MPAs in study area, a wide range of reef associated fish also undertake movements that are broader than the dimensions of the existing MPAs. Our sample is biased to the more highly mobile species of the reef fish community (snapper, porgies, jacks, grunts), and more work is required to include other key fish families not sampled or under-represented here, such as parrotfishes, wrasses and grouper.

### Time spent by fishes inside and outside MPAs in the U.S. Virgin Islands

Monitoring periods ranged from 1 to 967 days with high variance within and between species (Table 2). Few fish were detected for over 50% of the hours available during their monitoring period. French grunt (n = 1) and yellow goatfish (n = 3) exhibited the highest percentage time observed (81 & 64±.12%), followed by red hind (63%), and doctorfish (52±0.09%). The majority of fishes (7 out of 18 species) were present only 20-50% of the time and schoolmaster, nurse shark and dog snapper less than 20% of the time. Tagged fish exhibited high fidelity to VINP wherein they were initially captured and tagged. VINP represented over 70% of the time observed for 17 of the 18 fish species tagged. Fourteen percent of fishes (21 individuals of 6 species) tagged in VINP spent time outside of MPAs. Eight species spent 5–30% of their time inside VICR, with seven species not present at all and one species, queen triggerfish, present 61% of the time in VICR. Relatively little time was spent by fishes in the unprotected area outside of MPAs, with the highest being bar jacks (12%) and mutton snapper (5%).

**Table 2:** Summary data of percent time observed by species (total hours observed/total hours monitored (i.e., first to last detection) and the Fidelity Index score for VICR, VINP and non-MPA waters.

Family	Scientific name	Percent time observed (hrs present/hrs available)	Average Fidelity Index (± Stdev)		
			VICR	VINP	Outside MPA
Haemulidae	*Haemulon sciurus*	34±0.32	0.12±0.26	0.91±0.19	0.03±0.14
Lutjanidae	*Lutjanus synagris*	37±0.3	0.11±0.18	0.9±0.17	0.01±0.03
Sparidae	*Caranx ruber*	36±0.26	0.20±.25	0.85±0.23	0.02±0.06
Carangidae	*Calamus calamus*	49±0.31	0.06±0.11	0.91±0.2	0.12±0.25
Lutjanidae	*Ocyurus chrysurus*	41±0.3	0.01±0.03	0.98±0.05	0.02±0.08
Lutjanidae	*Lutjanus analis*	47±0.24	0.03±0.07	0.93±0.15	0.05±0.14
Holocentridae	*Holocentrus adscensionis*	38±0.38	0.14±0.37	0.93±0.19	0±0
Acanthuridae	*Acanthurus coeruleus*	39±0.27	0.01±0.02	0.99±0.01	0.01±0.02
Balistidae	*Balistes vetula*	30±0.24	0.61±0.54	0.39±0.54	0±0
Lutjanidae	*Lutjanus griseus*	39±0.17	0.33±0.4	0.7±0.35	0±0
Mullidae	*Mulloidichthys martinicus*	64±0.12	0.14±0.24	0.87±0.2	0.01±0.02
Acanthuridae	*Acanthurus chirurgus*	52±0.09	0±0	1±0	0±0
Serranidae	*Epinephelus guttatus*	63	0	1	0
Rhincodontidae	*Ginglymostoma cirratum*	22	0	1	0
Haemulidae	*Haemulon flavolineatum*	81	0	1	0
Haemulidae	*Haemulon plumierii*	13	0	1	0
Lutjanidae	*Lutjanus apodus*	0	0	1	0
Lutjanidae	*Lutjanus jocu*	11	0.06	0.94	0.04

The proportion of time represented in the FI ranged from 0 (no fidelity) to 1 (high fidelity).

Regardless of some extensive long distance movements and many shorter duration movements outside MPAs, the high fidelity to VINP and VICR of fishes tagged inside VINP indicates that on the south shore of St. John, these MPAs can be managed with confidence that many reef-associated fishes will likely experience the consequences of management actions. Most individuals, however, were also detected in the unprotected zone at some point during their tracking period. The time spent outside MPAs has greater uncertainty due to the inherent physical gaps in detection as a result of the design of our acoustic array. Our estimates of time spent outside MPAs should therefore be interpreted as a minimum estimate. Temporal gaps in detection during the total period of the study were most likely because of: 1) fish spending time in areas outside of the listening range of the receivers (further than 300 m or under coral heads or in noisy shallow water); or 2) fish moved outside of the study area and could have been outside of MPAs. Although unaccounted for in this study, it is possible that fish movements in and around highly structured coral reefs interfered with acoustic detectability and could explain the majority of unobserved time for reef-associated species such as grunts, squirrelfish, nurse shark and small snapper (i.e., schoolmaster, lane snapper). From a management efficacy perspective, however, if U.S. Virgin Islands MPAs can offer sufficient protection from fishing, and the boundaries and spawning aggregations are not heavily fished, then the high residence times recorded here suggest that these MPAs would offer adequate protection for many fishes.

### Potential scale mismatch with Caribbean MPAs

**Figure 5:**
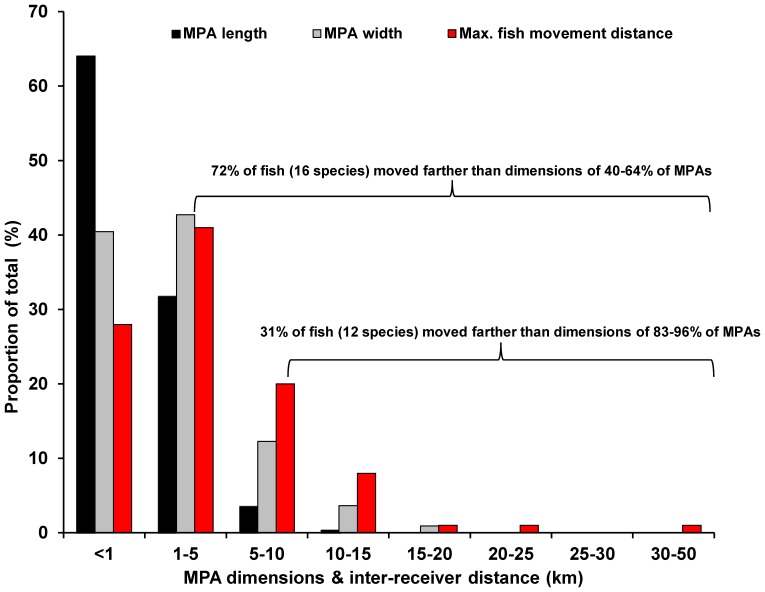
Histograms for the maximum spatial extent of movements for all tagged fish (n = 163) based on the least-cost pathway distance between the two farthest receivers with detections in the U.S. Caribbean; and the spatial dimensions of existing MPAs (n = 220) in the eastern Caribbean (only Leeward and Windward Islands).

According to the 2005 World Database on Protected Areas an estimated 40% of the protected areas in the global network of coral reef MPAs were smaller than 2 km^2^ [12]. Based on measurements of the spatial dimensions of 220 MPAs in the eastern Caribbean we reveal here that 85% are less than 5 km wide (median 1.35 km; range 0.04–21) and 70% are less than 5 km long (median 2.5 km; range 0.13–47.2 km). MPAs tend to be longer (along shore) than they are wide (shore to shelf) because they are sited primarily to protect the near-shore marine environments. Our comparison of distances moved by fish versus MPA size across the eastern Caribbean region revealed that 74% of our tracked fish (from 16 of 18 species) traversed distances (i.e. >1 km) greater than the dimensions of 40–64% of existing MPAs (Fig. 5). Twenty-eight percent of our fish (from 12 of 18 species) traversed distances broader than the dimensions of 69–85% of MPAs, emphasizing a potentially problematic scale mismatch between MPA design and the spatial scales of fish movements.

Many of the challenges encountered in managing populations of highly mobile animals occur because of a mismatch between the scale of management and the scale(s) of the ecological processes being managed [9], [10], [16]. Movement studies and data such as seafloor maps can be used to assess if an MPA encompasses the routine and critical spaces (i.e., nursery, spawning) used during the life-history of highly mobile marine fauna. Re-scaling MPAs to more fully encompass ecological neighborhoods for a group of target species could make major advances in achieving conservation goals. Alternatively, if MPA boundaries can be delineated along physical features (i.e., expanses of unsuitable habitat) that function as natural barriers to fish movement then perhaps even relatively small marine reserves would offer enhanced protection through de-facto containment of reef-associated fish [41], [42], [44]. On small tropical islands, designing MPAs that extend across the insular shelf would better protect inshore-offshore migration corridors, thus encompassing the critical habitats within the life-cycle. For fishes vulnerable to fishing, but with well-defined linear movement routes, design of MPAs that include key migration pathways, so called ‘blue corridors’, connecting essential habitat areas will likely result in greater protection. Terrestrial conservation planning for highly mobile populations routinely consider connectivity and incorporate movement corridors in protected area design, although efficacy of corridors is not well tested [27].

To some extent, the expansion of two no-take MPAs in the U.S. Virgin Islands in 2003 to encompass some of the adjacent deeper water coral reefs, improved the likelihood that fishes undertaking onshore-offshore ontogenetic shifts or migrations will receive protection from fishing. Further research is needed to examine the shape of MPAs relative to their placement across the shelf in order to assess the level of protection provided to fishes that undergo cross-shelf movements (i.e. nearshore reefs to deeper offshore waters) through ontogenetic habitat shifts and spawning migrations. Conversely, if the objective is to enhance spillover then MPA placement in an area where the habitat inside is spatially connected to suitable habitat outside would enhance emigration of fish biomass [19]. Little is known about the influence of MPA placement and shape relative to seascape patterning, fish population response and MPA performance. Comparative studies of fish movements across multiple MPAs of different sizes protecting different spatial configurations of coral reef are required to address this knowledge gap.

It is important to note that our database of Caribbean MPAs did not indicate which MPAs (or portions of MPAs) were no-take areas/fishery closures. We also did not account for the level of protection offered by individual MPAs or the fact that MPAs could be adjacent or overlapping another MPA therefore providing greater spatial coverage in protection. This level of information has not yet been compiled for the entire Caribbean region, but it is likely that most MPAs are multi-use and open to fishing. No-take areas in the Caribbean are typically smaller than multi-use MPAs due to the socio-economic impact of restricting fishing on small island developing states. If true, this would mean that the scale mismatch between no-take areas and the spatial scale of fish movement could be an even greater disparity than highlighted here possibly resulting in insufficient protection to achieve the expected role in replenishing fished populations and rebuilding sustainable local fisheries.

### Implications for ecologists

Rarely do fish ecologists measure habitat structure at spatial scales relevant to organism movements [9]. Based on the spatial movements revealed by our study, sampling and quantifying habitat correlates only with conventional survey techniques such as underwater transect lines and quadrats (10 s–100 s m^2^) appears inadequate. Such fine-scale measurements provide only a snapshot of habitat association and potentially result in identification of misleading environmental drivers. In light of the greater mobility of reef fish demonstrated here and an acknowledgement that many fish use mosaics of habitat types, we advocate that the quantification of fish-habitat requires a conceptual shift to a multi-scale, multi-habitat, organism-centered approach analogous to the recent growth of landscape ecology to study mobile terrestrial fauna [28]–[30]. Several guiding principles that exist at the core of landscape ecology have made major contributions to terrestrial landscape planning and conservation, but in marine systems our understanding is still in its infancy. For example, (1) environmental heterogeneity exists at multiple spatial scales to which organisms respond differently and at different scales; (2) connectivity is an important ecological pattern and process; (3) patch boundaries/edges influence ecological processes. In landscape ecology, the organism's activity space helps us to anchor the scale selection to a known and functionally meaningful spatiotemporal scale that can then form the focal scale in a hierarchical approach [9]. The integration of concepts from behavioral ecology and landscape ecology represents a new frontier in marine ecology and offer many opportunities for scientific progress together with providing support for information-led design in marine management [31]. The ecological importance of adult movements is receiving renewed attention and it has been argued that information on adult-mediated population connectivity is critical to understanding the complex dynamics of connectivity between subpopulations, patterns of recruitment and the evolution of population structuring [37]. If movements of adult fishes play a large role in regulating regional patterns in productivity then this has important implications for fisheries management and the design and functioning of place-based strategies such as MPAs. Technological advances in microelectronics for telemetry, spatial analytical techniques and marine remote sensing now offer an unprecedented opportunity to address major knowledge gaps in marine animal movements for a wide range of mobile marine organisms.

## Supporting Information

File S1 (DOC)
