# Supplementary material for: Fish with Chips: Tracking Reef Fish Movements to Evaluate Size and Connectivity of Caribbean Marine Protected Areas
Source: PLoS One. 2014 May 5;9(5):e96028. doi: 10.1371/journal.pone.0096028 (PMC4010402; doi:10.1371/journal.pone.0096028)
Supplement: File S1 — (DOC) [file pone.0096028.s001.doc]

**S1 Supplementary Information:**

**Pittman et al.**

**Materials and Methods:**

*Acoustic tagging*

Fish were captured using baited fish traps and hook and line under permit within the VINP. Fish were transported in aerated tubs and hypodermic needles were used to release gas from the swim bladders of fish which showed signs of barotrauma. All fish were transported to a 450 gallon shore-based holding tank with flow-through seawater at a rate of 23 liters/minute.

We surgically implanted VEMCO V9-2L-R64K transmitters (9 mm x 29 mm, 2.9 g in water) into the stomach cavities of captured fish. These transmitters periodically emit a ‘pulse train’ of closely spaced 69-kHz ‘pings’, which uniquely identify each fish. These pulse trains are a few seconds in length, and the transmitters are silent for a randomized period between each pulse train. Each successfully decoded pulse train is recorded as a single detection by a VR2 receiver, and is stored in the receiver memory as the unique transmitter number, date and time of detection. The tag manufacturers estimated maximum battery life is 738 days, however, our longest duration for a tagged fish between first and last detection was 967 days.

Acoustic transmitters were coated in a combination of beeswax and paraffin (1:2.33) to reduce immuno-rejection. A 1 cm incision was made 1cm off-center from the ventral midline behind the pelvic fins and the V9 acoustic transmitter was placed within the body cavity. The incision was closed with two surgical sutures and the fish were observed in a holding tank to ensure adequate recovery. After 24 hours in post-surgery holding tanks, fish were released at a location in close proximity to their original capture location. Queen triggerfish (*B. vetula*) and surgeonfish (Acanthuridae) were unsuitable for surgical implantation because of a laterally compressed body shape and small stomach cavity. These species were tagged externally by gluing transmitters to small (1 cm) disk tags with steel pins and inserting the pins through the dorsal musculature or behind the caudal fin. Although fish from 18 species were tracked, five species of snapper (Lutjanidae) accounted for 31 % of all individuals tagged and three species of grunt (Haemulidae) accounted for 23 % of the total. The tracking data are archived by NOAA Center for Coastal Monitoring and Assessment.

*Spatial analysis to calculate least-cost distance between receivers*

Distances between all fixed hydrophones in the array were computed within a Geographic Information System (ESRI ArcGIS v10.1) using a spatial analysis tool called the Cost Path function to provide the least cost pathway (LCP). We use this tool to calculate the path across the seascape that has the minimum accumulative travel cost. First a cost surface is modeled by assigning cost values to raster grid cells across the seascape. In this case, highest cost was assigned to cells with terrestrial land (cost of 1,000,000) and for waters beyond the shelf edge (cost of 1,000) and lowest cost was assigned to map cells where coral reef communities exist across the shelf (sand = 10; coral reefs = 1). As a result, the pathways of least cost are conditioned to follow preferred habitat structure (i.e., coral reef communities), avoiding terrestrial land and deep water. Large expanses of sand have been found to function as barriers to movement for some reef fish, but low cost here allowed pathways to cross sand in order to link the even lower cost reef habitat patches. In addition, the species tagged in this study are rarely seen over sandy sediments remote from coral reefs during daytime underwater surveys (personal observations from all authors based on more than 10,000 surveys). Little is known, however, about movements at night. The tool was implemented through a customized Python toolbox that calculates the least cost paths among an array of points given a provided cost surface and builds a distance matrix that summarizes these least cost paths. The distances between all receivers visited by an individual fish was then extracted to find the greatest distance traveled. The least cost pathway code is available upon request from the authors and may be implemented for any set of points and corresponding cost surface. Seafloor structure was classified from an integration of best-available bathymetry and benthic habitat maps available online at:

<http://ccma.nos.noaa.gov/ecosystems/coralreef/usvi_nps.aspx>
